# From a Runny Nose to a Slow Heart: A Case Report About Disentangling the Mystery of A Heart Block Secondary to Influenza

**DOI:** 10.7759/cureus.85075

**Published:** 2025-05-30

**Authors:** Sabavath Arun, Shivangi Sinha, Kundavaram Rajkumar, Amber Kumar, Shikha Malik

**Affiliations:** 1 Pediatrics, All India Institute of Medical Sciences, Bhopal, Bhopal, IND; 2 Pediatrics, All India Institute of Medical Sciences, Raebareli, Raebareli, IND

**Keywords:** atrioventricular heart block, atypical pneumonia, dexamethasone, h1n1 influenza, myocarditis

## Abstract

Respiratory tract infections are frequently seen in children, with influenza responsible for the majority of hospital visits. While the respiratory manifestations of influenza are well recognized and managed by pediatricians, cardiovascular complications such as myocarditis, pericarditis, and conduction abnormalities are less commonly encountered and often overlooked. These complications, though rare, can have serious clinical implications if not promptly identified and treated. Here we present a rare case of influenza-induced third-degree heart block, which responded to dexamethasone therapy. A seven-month-old male child presented with fever, cough, rhinorrhea, and increased work of breathing. The child was initially managed as a case of pneumonia with intravenous antibiotics and supportive therapy. The findings from a chest radiograph were suggestive of viral pneumonia. With negative blood cultures and normal inflammatory markers, antibiotics were discontinued. Hemagglutinin Type 1 and Neuraminidase Type 1 (H1N1) influenza was confirmed on reverse transcription polymerase chain reaction (RT-PCR) testing. Though there was an improvement in the respiratory signs and symptoms, the child developed bradycardia with hypotension. Electrocardiography revealed a third-degree atrioventricular block with ST-T changes, normal cardiac structure, and preserved left ventricular function. Cardiac biomarkers were within normal limits. Despite the initiation of isoprenaline for persistent bradycardia, no improvement was seen. A short course of dexamethasone was administered, following which the child’s cardiac rhythm reverted to the normal sinus rhythm. This case underscores the importance of considering cardiovascular complications, like conduction abnormalities, in children presenting with an influenza-like illness. Early recognition and timely corticosteroid therapy might restore normal cardiac conduction and potentially prevent the need for invasive interventions like the placement of a pacemaker.

## Introduction

The influenza virus, belonging to the family Orthomyxoviridae, is an enveloped, segmented, negative-sense, single-stranded RNA virus [1]. This enveloped virus is an important public health problem globally and is responsible for three to five million cases of severe illness and approximately 300,000 deaths annually [2]. While its respiratory manifestations, such as fever, cough, and nasal congestion, are well known, influenza can also lead to less common but serious complications, including those affecting the cardiovascular system [3]. Among these are myocarditis, pericarditis, and conduction abnormalities, including arrhythmias and atrioventricular (AV) blocks [4]. Though rare, influenza-induced high-degree AV block can be life-threatening, especially in pediatric patients. Hence, timely recognition and management are crucial. This case report presents a rare instance of third-degree heart block in a seven-month-old child with Hemagglutinin Type 1 and Neuraminidase Type 1 (H1N1) influenza, which responded favorably to a short course of steroids after the failure of standard medical therapy. This approach avoided the need for pacing in the resource-limited setting.

## Case presentation

**Figure 1:**
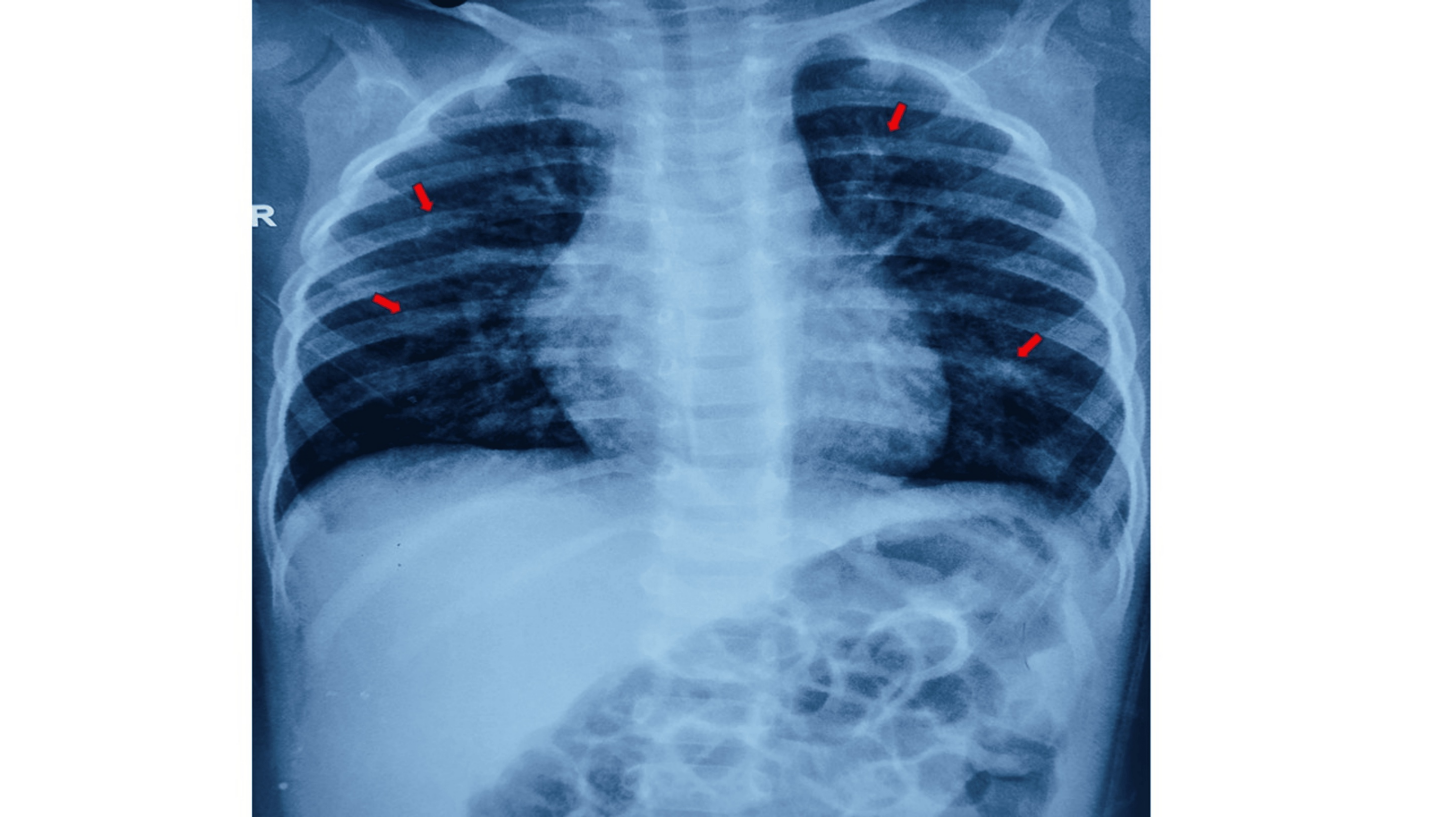
Chest X-ray (supine film) of the patient Chest radiograph showing bilateral, multiple, heterogenous opacities (red arrows).

A seven-month-old male child presented with complaints of fever, a runny nose, and cough for three days, and fast breathing for a day. On initial impression, the child had signs of increased work of breathing and supportive care was promptly initiated. Blood gas revealed mild hypoxemia, and the chest radiograph showed bilateral, multiple, heterogenous opacities (Figure 1).

Empirical antibiotic treatment was started and respiratory support in the form of continuous positive airway pressure was continued. Given the findings from the chest radiograph, atypical pneumonia (more likely viral) was considered and oseltamivir was initiated. The respiratory distress improved and the fever spikes responded during hospitalization. The laboratory workup of the child is summarized in Table 1.

**Table 1:** The patient's laboratory work up H1N1: Hemagglutinin Type 1 and Neuraminidase Type 1; RT-PCR: Reverse Transcription Polymerase Chain Reaction; T4: Thyroid hormone T4; CK-MB: Creatine kinase-MB;  pro-BNP: pro B-type natriuretic peptide

Parameter	Observed	Reference range
Hemoglobin (gm/dL)	10.6	11-15
Total leucocyte count (cells/microL)	7080	4000-11000
Neutrophils (%)	31	40-70
Lymphocytes (%)	59	20-40
Eosinophils (%)	3%	1-6
Monocytes (%)	7	2-8
Platelet count (X 10^5^/microL)	2.56	1.5-4.5
Mean corpuscular volume (fL)	89	76-93
Mean corpuscular hemoglobin concentration (g/dL)	31.6	32-36
Mean corpuscular hemoglobin (pg)	32.8	27-32
Urea (mg/dL)	30	20-40
Creatinine (mg/dL)	0.3	0.2-0.7
Sodium (mEq/L)	135	135-145
Potassium (mEq/L)	4.2	3.5-5.5
Chloride (mEq/L)	104	98-107
H1N1 RT-PCR	Positive	-
Thyroid Stimulating Hormone (microU/mL)	1.5	0.4-4.94
Free T4 (ng/dL)	1.35	0.7-1.48
Procalcitonin (ng/mL)	0.1	<0.2
Troponin T and I	Negative	-
CK-MB (IU/L)	9	<35
Pro-BNP	Negative	-
Blood culture	Sterile	-
Respiratory syncytial virus PCR	Negative	-
COVID RT-PCR	Negative	-
Para-influenzae RT-PCR	Negative	-
Human Adenovirus RT-PCR	Negative	-
Human Metapneumovirus RT-PCR	Negative	-
Human Enterovirus RT-PCR	Negative	-

The child developed bradycardia on the second day of hospital admission with a maintained hemodynamic status. All the reversible causes of bradycardia were ruled out and the 12-lead electrocardiogram (ECG) showed a third-degree heart block with non-specific ST-T changes (Figure 1).

**Figure 2:**
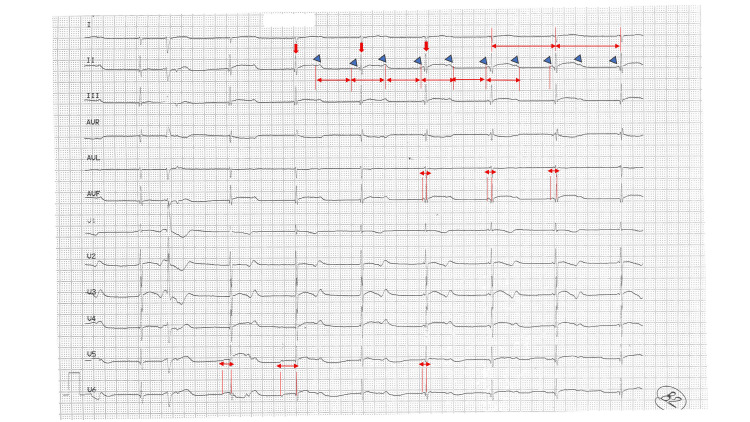
Twelve lead electrocardiogram of the patient AV: Atrioventricular dissociation; AVF: Augmented vector foot Lead II shows a constant RR interval and constant PP interval. Vertical red arrows show R waves, and the blue arrow heads show P waves with AV dissociation, and a ventricular rate of 50 and an atrial rate of 100. Lead AVF and V6 show PR variability, confirming an advanced heart block.

A provisional diagnosis of viral myocarditis was considered and work up was done. Troponin I, troponin T, creatinine kinase (CK-MB), pro-brain natriuretic peptide (pro-BNP), and 2D echocardiography were within normal limits. With the background of H1N1 pneumonia along with bradycardia, after ruling out other causes, a possibility of Influenza-induced conduction abnormality was considered. 

The child was initially monitored for spontaneous reversal of rate and rhythm, but he had persistent bradycardia (though without any hemodynamic compromise initially). On day five of the hospital stay, the patient had hemodynamic worsening in the form of decreased blood pressure and prolonged capillary refill time. Isoprenaline was initiated with no clinical response. In view of clinical deterioration despite isoprenaline, the administration of a short course of dexamethasone was considered (0.15mg/m^2^ of dexamethasone was given every six hours for a total of six doses). This intervention dramatically restored the patient's rate and rhythm to normalcy. The child was discharged after eight days of hospital stay and was followed up in the pediatric cardiology OPD. Follow-up office visits showed no recurrence of the rhythm abnormality and echocardiography also revealed normal function. 

## Discussion

Clinical manifestations of influenza vary from asymptomatic, mild respiratory symptoms (sore throat, fever), to dreaded cardiovascular complications like thromboembolism and myocarditis. The incidence of cardiovascular complications associated with the influenza virus has been reported up to 1.51%-17.47% [4]. In a systematic review conducted by Ouranos et al., the most frequent cardiovascular manifestations were myocardial infarction (MI), myocarditis, pericarditis, and pancarditis [5]. There are a few cases of influenza patients with conduction defects reported in the literature [6-9], but the number remains sparse. Conduction defects associated with influenza infection were documented a century ago for the first time by Cockayne, with the majority being bradycardia and low-grade AV block [10]. With the ever-changing and evolving knowledge, various arrhythmias associated with influenza infection have also been reported, including atrial and ventricular fibrillation [5]. 

Though the precise pathogenesis of the AV block associated with influenza infection remains elusive, the proposed mechanisms include direct viral damage to myocardium and vessels and a systemic inflammatory response induced by infection. The influenza virus enters the epithelial cells of the pulmonary alveoli and the myocardium, and starts an immune signaling cascade, including the induction of cytokines, leading to both cellular and humoral immune responses. Along with this, influenza has been shown to have direct inflammatory effects on arteries and atherosclerotic plaques, leading to plaque accumulation and instability. This ultimately results in a potential MI, myocardial injury, and cardiac dysfunction. The systemic response to the influenza infection is marked by the activation of various cytokines, which can lead to plaque destabilization, resembling that seen in an MI [5,11]. 

Viral invasion can result in edema and cellular infiltration of the AV node, leading to conduction disturbances, whereas a severe influenza infection leads to a cytokine storm, with elevated levels of tumor necrosis factor (TNF-α), IL-1, and IL-6. These inflammatory mediators can alter ion channel function and disrupt normal conduction. They may also induce fibrosis or apoptosis of the conduction cells in the AV node. Whether these two mechanisms work in tandem to increase the risk of cardiovascular complications is currently debated [3].

A thorough literature review suggests the initial use of medical therapy for conduction abnormalities secondary to infective etiologies. This medical management includes the use of beta agonists, atropine etc. A temporary pacemaker can be considered in patients with second-degree or third-degree AV block and those with symptoms of hemodynamic compromise refractory to medical therapy [11]. In our case, the rhythm abnormalities persisted even after the resolution of respiratory symptoms, later leading to a hemodynamic compromise. Appropriate response was not noted despite starting isoprenaline, so steroid therapy was started while considering a possible inflammatory response. The cardiac rate and rhythm reverted to normal after a short course of steroids. Most of the previously reported cases were managed with the placement of a pacemaker, and a majority of them responded to this approach [12]. In our case, we managed the AV block with a short course of steroids, although the evidence for the role of steroids in the same has been scarcely reported [12]. 

## Conclusions

This is one of the few cases where an advanced heart block was reversed without the use of a pacemaker. This case highlights the rare but notable association between influenza infections, particularly H1N1, and the development of transient arrhythmias, underscoring the importance of vigilant monitoring and timely intervention in managing such cases. While further research is needed to better define the role of steroids and other therapies in managing influenza-associated conduction abnormalities, this case emphasizes the critical need for early recognition, careful monitoring, and tailored interventions. Understanding the multifaceted impacts of influenza on the cardiovascular system is crucial for improving patient outcomes and advancing our knowledge of viral-induced arrhythmias.
